# Quiescent innate and adaptive immune responses maintain the long-term integrity of corneal endothelium reconstituted through allogeneic cell injection therapy

**DOI:** 10.1038/s41598-022-22522-4

**Published:** 2022-10-27

**Authors:** Munetoyo Toda, Morio Ueno, Jun Yamada, Asako Hiraga, Kazuko Asada, Junji Hamuro, Chie Sotozono, Shigeru Kinoshita

**Affiliations:** 1 Department of Frontier Medical Science and Technology for Ophthalmology, Kyoto Prefectural University of Medicine, Kyoto, Japan; 2 Department of Ophthalmology, Kyoto Prefectural University of Medicine, 465 Kajii-Cho, Hirokoji-Agaru, Kawaramachi-Dori, Kamigyo-Ku, Kyoto, 602-0841 Japan

**Keywords:** Medical research, Translational research, Corneal diseases

## Abstract

This study aims to clarify the immunogenicity in acquired and innate immune responses of cultured human corneal endothelial cells (hCECs) applied for cell injection therapy, a newly established modality for corneal endothelium failures. Thirty-four patients with corneal endothelial failure received injection of allogeneic hCEC suspension into anterior chamber. No sign of immunological rejection was observed in all 34 patients during the 5–8 years postoperative follow-up period. Cell injection therapy was successful in 2 patients treated for endothelial failure after penetrating keratoplasty and one patient with Descemet membrane stripping automated endothelial keratoplasty failure. ELISPOT assays performed in allo-mixed lymphocyte reaction to the alloantigen identical to that on the injected hCECs, elicited sparse IFN-γ-specific spots in the peripheral blood mononuclear cells of patients who received hCEC injection. The therapy generated simple and smooth graft-host junctions without wound stress. The injection of C57BL/6 CECs into the anterior chamber of BALB/c mice, which rejected C57BL/6 corneas 6 weeks ago, induced no sign of inflammatory reactions after the second challenge of alloantigen. Collectively, injection of the hCEC cell suspension in the aqueous humor induces immune tolerance that contributes to the survival of the reconstituted endothelium.

## Introduction

Allogeneic corneal endothelial transplantation, or keratoplasty, is currently the standard clinical modality for patients with corneal endothelial failures. With this advanced tissue transplantation technique, the clinical problem of endothelial graft rejection seems to be largely improved. In fact, the immunological rejection of Descemet’s membrane endothelial keratoplasty (DMEK) transplants from donors is a very rare event in current practice (< 5% of patients with a 15 years follow-up)^1^. However, chronic endothelial functional failure remains a highly serious issue that is still elusive to overcome^2–8^.

We have proposed a new surgical modality to reconstitute the failed human corneal endothelium (HCE) by injecting cultured HCE cells (hCEC) into the anterior chamber (AC)^9,10^. We have identified hCEC subpopulations (SPs) with high clinical efficacy (occasionally described as effector cells)^10,11^ in regard to the rapid recovery of central cornea thickness (CCT), better corneal endothelial cell density (CECD) and low CECD attrition over 5 years post-surgery.

One possible adverse effect of cell injection therapy, in regard to the allogenic immune reaction, is an aberration due to the delivery of the injected cells to other organs. In this context, we previously evaluated the distribution of the injected hCECs in monkey organs and in cryogenically-injured corneal endothelium mouse-models, and found that the presence of bare Descemet’s membrane after corneal endothelial damage is responsible for the selective scavenging of infused hCECs mostly on damaged corneal endothelium with no aberrant ectopic cell presence.

In cell injection therapy, hCECs from one donor derived cells can be injected to a minimum of 20 recipients^9–14^. Under these circumstances, the immune phenotype of produced hCECs becomes a serious issue, considering the wide scope of this modality against highly populated Fuchs endothelial corneal dystrophy (FECD) patients. This has prompted us to confirm the safety, both in clinical settings and in basic model systems, of this new modality from the viewpoint of the immune–inflammatory axis.

An hCEC injection could involve a lower risk for graft rejection in comparison to penetrating keratoplasty (PK), as the corneal endothelium (CE) was shown to be less involved in allogeneic immunity in contrast to corneal stromal and epithelial tissue^15,16^. CE may contribute to maintaining the privileged immune status of the AC by inducing peripheral immune tolerance^17–19^. The ocular immune privilege is maintained by regulating the innate and adaptive immune response, in addition to immunosuppressive microenvironments of the ocular system, such as diverse mediators in AC^20–23^. Recently, we reported that immune tolerance occurs after infusion of murine allogeneic donor-derived endothelial cells into the AC, in addition to the induction of anterior chamber-associated immune deviation (ACAID)^24^.

Here, we present the results of an ELISPOT assay in our Phase 2 and 3 clinical trials that aimed to elucidate whether antigen recognition of the injected hCEC cells would occur in patients who received our cell injection therapy. It is quite intriguing that hCEC injection cells could successfully survive, even in patients with a history of failed corneal grafts.

## Results

### No failure of reconstituted HCE in patients injected with hCECs

**Figure 1:**
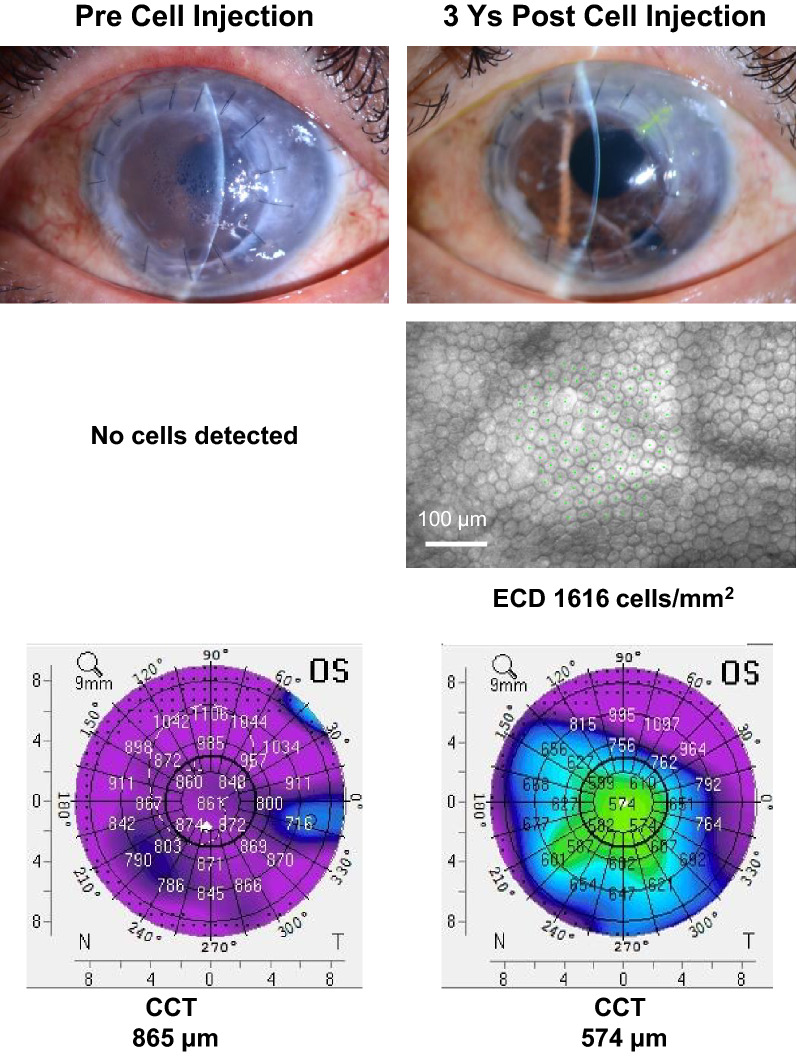
Pre- and post-cell injection therapy images (slit-lamp, specular microscopy and Scheimpflug) of a patient with failure of previous keratoplasty. He underwent penetrating keratoplasty (PK) in 2001 for keratoconus in his left eye and received the second PK in May 2014 for corneal graft failure caused by graft rejection. Subsequently, he underwent non-Descemet stripping automated endothelial keratoplasty (nDSAEK) in May 2016 for graft failure. Although the nDSAEK graft was in good condition at 4 months postoperative, graft failure occurred at 9 months postoperative, resulting in the severe impairment of best corrected visual acuity (BCVA). These images were obtained at pre-cell injection in March 2017 (left) and 3 years post-cell injection in March 2020 (right). Scale bar: 100 μm.

A first-in-human clinical trial was performed in 2013, when three graft failure patients were included in our clinical research trial. Figure 1 shows the slit-lamp microscopy images (upper), specular microscopic images (middle) and Scheimpflug images (lower) of patient 33, who received cell injections, obtained prior to and after surgery. Although this patient had PK twice and a DSAEK treatment, no sign of immunological rejection was observed three and a half years after the hCEC injection. We are continuing to follow up on 21 patients who received hCEC injection therapy, but we have not found any signs of immunological rejection in any patients (Table 1). These clinical outcomes suggest that antigen recognition of the injected cells would rarely or never occur in our newly proposed therapy.

**Table 1:** Pre- and post-operative clinical summary of the 21 patients.

Patient No	Disease subtype	Postoperative period (years)	Rejection
1	ALI-CEF	7.83	–
2	FECD	7.92	–
3	FECD	5.00	–
4	PEX-CEF	7.50	–
5	FECD	4.92	–
6	ALI-CEF	7.58	–
7	FECD	7.33	–
8	FECD	7.33	–
9	IOS-CEF	5.00	–
10	FECD	7.00	–
11	FECD	7.17	–
12	FECD	6.50	–
13	ALI-CEF	6.50	–
14	GF	6.50	–
15	ALI-CEF	6.50	–
16	PCEF	6.33	–
17	GF	5.92	–
18	CHED	6.42	–
32	ALI-CEF	2.75	–
33	GF	5.00	–
34	PCEF	4.92	–

In this study, corneal endothelial failure was clinically diagnosed under ophthalmic examination. The definition was as follows: (1) the appearance of endothelial rejection lines or many precipitates at the posterior corneal surface with ciliary injection, (2) corneal epithelial and stromal edema either at the regional or whole cornea, and (3) apparently decreased best-corrected visual acuity. No graft failure was observed in all patients during the 5–8 years post-operative follow-up period.

*ALI-CEF* Argon laser iridotomy-induced corneal endothelial failure, *CHED* Congenital hereditary endothelial dystrophy, *FECD* Fuchs endothelial corneal dystrophy, *GF* Graft failure, *IOS-CEF* Intraocular surgery-related CEF, *PCEF* Pseudophakic CEF, *PEX-CEF* Pseudoexfoliation syndrome-related CEF.

### No induction of IFN-γ against injected allogeneic hCECs

**Figure 2:**
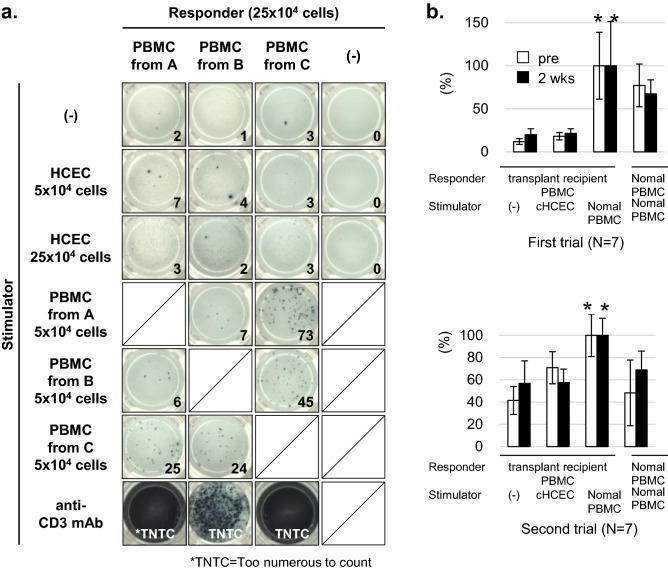
CD4+ dependent immune reaction against hCECs. (**a**) PBMCs from 3 healthy donors (A, B and C) were stimulated with hCECs or PBMCs from other healthy donors (allo-PBMCs) at the ratio of 5:1 or 1:1. After incubation for 2 days, IFN-γ production was analyzed by ELISPOT assay. Stimulation with anti-CD3 antibody was used as the experimental positive control (lower panel). The numbers of detected spots were shown in each panel (TNTC = Too numerous to count). (**b**) PBMCs from the patients were stimulated with the same lot of hCECs injected at the S/R ratio of 1:5. Seven patients in the first trial group and ten patients in the second trial group were examined. Normalized data with allo-MLR are shown. (Student’s paired t-test *: *p* < 0.001). Further, Kruskal–Wallis test was used and post‐hoc analysis with Steel’s multiple comparison test were performed. (Upper panel) (Pre): Kruskal–Wallis test among three groups; normal, PBMC (-), cHCEC, *p* < 0.001, Steel's multiple comparison test; normal versus PBMC (-) *p* = 0.003, normal versus cHCEC, *p* = 0.003 (2 weeks): Kruskal–Wallis test among the same three groups, *p* = 0.027, Steel's multiple comparison test; normal versus PBMC (-) *p* = 0.033, normal versus cHCEC, *p* = 0.047. (Lower panel) (Pre): Kruskal–Wallis test among three groups; normal, PBMC (-), cHCEC, *p* < 0.061, Steel's multiple comparison test; normal versus PBMC (-) *p* = 0.055, normal versus cHCEC, *p* = 0.446 (2 weeks): Kruskal–Wallis test among the same three groups, *p* = 0.103, Steel's multiple comparison test; normal versus PBMC(-) *p* = 0.064, normal versus cHCEC, *p* = 0.242.

Subsequently, we examined the CD4^+^ dependent immune reaction against injected hCECs by monitoring the IFN-γ production in the mixed lymphocytes reaction (MLR). An ELISPOT assay for IFN-γ was applied. IFN-γ-specific spots were clearly detected at an S/R ratio of 1/5 in the control alloMLR groups (combination of allo-PBMCs), whereas few spots were detected even at an S/R ratio of 1/1 in the hCECs/PBMCs groups (Fig. 2a). Two weeks after the hCEC injection, PBMCs from the patients were further stimulated with hCECs; the same group of hCECs was injected at the S/R ratio of 1:5. As shown in Fig. 2b, there were significantly fewer IFN-γ-specific spots in PBMC obtained from patients who received hCEC injections than in the control allo-MLR group.

### Expression of immunomodulatory molecules on cHCECs

Apart from the microenvironmental milieu in aqueous humor (AH), we had wondered about the possibility of the distinct expression of immunomodulatory molecules between hCEC subpopulations (SPs) with a higher and a lower clinical efficacy. PD-L1 and GITRL (although marginally expressed) were expressed on both SPs (Supplementary Fig. 1a). CD40L, TSP-1, CTLA4, TGF-RII and, unexpectedly, FASL were not expressed. Additionally, the expression of surface HLA class I antigen was elevated on hCEC SPs with low clinical efficacy compared to those with higher clinical efficacy (Supplementary Fig. 1b).

### Delayed-type hypersensitivity response to allogeneic antigens in mpCEC-injected mice

**Figure 3:**
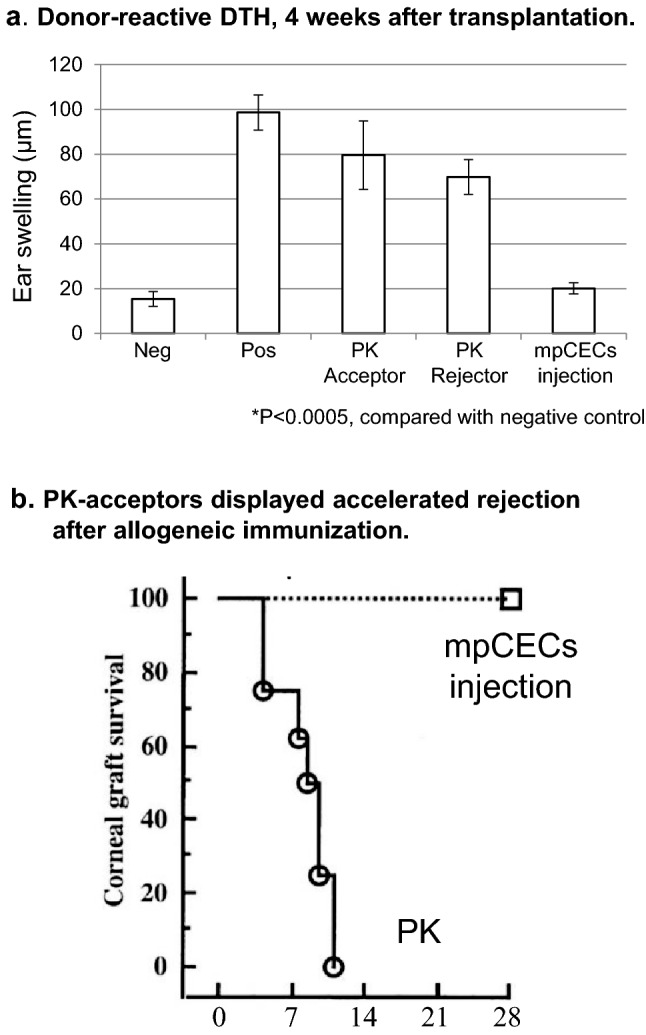
(**a**) Donor-reactive DTH 4 weeks after allogeneic corneal penetrating keratoplasty (PK) or cell injection. One group of BALB/c mice received C57BL/6 corneal penetrating keratoplasty (PK, N = 17), and another group of BALB/c mice received mouse primary corneal endothelial cell injection (mpCECs injection, N = 11). Four weeks later, they were ear-challenged with donor-derived splenocytes, and swelling responses were assessed 24 h later. Naive mice were used as a negative control, and mice subcutaneously immunized with donor splenocytes were used as a positive control. *Indicates responses significantly higher than those of the control group (Student’s paired t-test, *p* < 0.005). Further, Kruskal–Wallis test was used and post-hoc analysis with Steel's multiple comparison test were performed. Kruskal–Wallis test among five groups; negative control, positive control, PK acceptor, PK rejector and mCEC transplantation, *p* < 0.001, Steel's multiple comparison test; negative versus positive control *p* = 0.032, negative control versus PK acceptor *p* = 0.032, negative control versus PK rejector *p* = 0.031, negative control versus mCEC transplantation *p* = 0.458. (**b**) Systemic allo-sensitization was performed by injecting C57BL/6 splenocytes subcutaneously either into pre-PK treated or mpCEC-injected BALB/c mice. The grafted cornea tissues were promptly rejected in all mice in the pre-PK treated mice (N = 8), whereas in the hCEC pre-injected mice, the reconstituted CECs were not rejected, and their corneas remained clear and stable.

To determine whether recipients would be sensitized by alloantigens on the hCECs injected, we compared the induction of delayed-type hypersensitivity (DTH) between corneal penetrating keratoplasty (PK) and mpCEC injection in murine models. One group of BALB/c mice received C57BL/6 corneal penetrating keratoplasty allografts (PK, N = 17), and another group of BALB/c mice received mouse primary corneal endothelial cell injection (mpCECs injection, N = 11). In PK-treated mice, DTH was positive at 4 weeks after transplantation in both acceptors and rejectors of allogeneic corneas, while in mpCEC-injected mice, DTH was almost comparable with the negative control (N = 5 each) (Fig. 3a).

### AC-injected mpCECs were not rejected in alloantigen-pre-sensitized mice

To understand the mechanism underlying the intriguing clinical finding that patient #33, who rejected allografts twice in PK and once in Descemet membrane stripping automated endothelial keratoplasty (DSAEK) before enrollment in hCEC injection clinical research, elicited no sign of immunological rejection of reconstituted CEC at three-and-a-half years after hCEC injection surgery, we were tempted to analyze the mechanism also in mice allograft models. We allo-sensitized recipient mice systemically by injecting C57BL/6 splenocytes subcutaneously either into pre-PK treated or mpCEC-injected BALB/c mice. The grafted cornea tissues were promptly rejected in all mice in the pre-PK treated mice (N = 8), whereas in the hCEC pre-injected mice, the reconstituted CECs were not rejected, and their corneas remained clear and stable (Fig. 3b). These results suggest that hCECs injected into AC were not rejected, even when the recipient was pre-sensitized by allogeneic antigens.

Furthermore, we performed the injection of C57BL/6-derived mpCECs into PK rejector C57BL/6 mice (n = 5) at 6 weeks post PK. All mpCECs injected eyes showed no sign of inflammatory reactions or opacity associated with rejection 2 weeks after the injection of mpCECs (data not shown).

### The cytokine profiles in the sera

**Figure 4:**
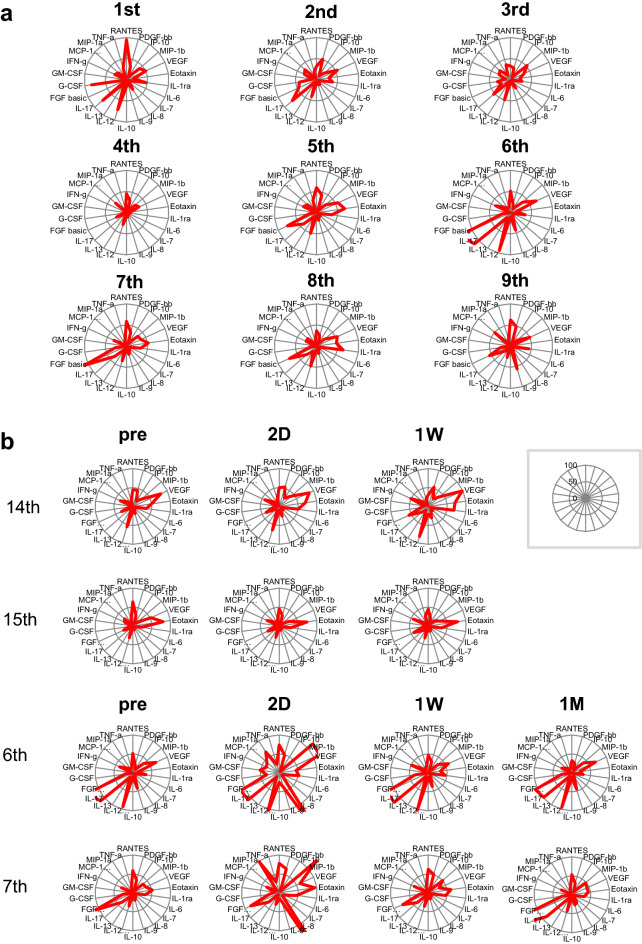
Host response after cell injection. (**a**) Sera from 9 patients in the clinical study were collected on the day before surgery and analyzed using Bio-Plex. (**b**) Patients’ sera injected with a relatively lower proportion (56%) of mature hCEC SPs (patients 6 and 7) or a relatively higher proportion (> 90%) (patients 14 and 15) were analyzed using Bio-Plex.

The cytokine profiles in the sera of nine patients who received hCEC injection therapy in clinical research (first in human to 9th patients) were harvested 1 day before cell infusion surgery to know the background levels of 27 cytokines in sera to distinguish the changes of cytokines after hCEC injection into AC firstly performed in the world. The cytokine profiles of each patient were analyzed using the Bio-Plex Human 27-Plex Panel Kit and expressed as a radar chart (Fig. 4a). Some patients showed the high levels in VEGF, Eotaxin and FGF-b, but others did not, indicating the presence of profile diversity among patients. Next, to know whether the cell injection would cause the variation of cytokine profiles in sera, levels of 27 cytokines in sera of four patients before and after cell infusion were analyzed similarly. The patients (6th and 7th) injected with hCEC with a relatively lower proportion of mature hCEC Sps^11^ (~ 75%) showed transient but marked increase in some cytokines 2 days after hCEC injection than the patients (14th and 15th) injected with hCEC Sps with a relatively higher proportion of mature hCEC Sps^11^ (> 90%) (Fig. 4b). This indicates the quality of infused hCEC might, albeit transiently, influence the production of inflammatory cytokines such as TNF-α, IL-8 and MIP-1β (Fig. 4b).

### Cytokine profiles in pre-surgical aqueous humor

Considering that a certain class of cytokines were elevated in the AH of BK patients (Supplementary Table 1), it may be possible that distinctive cytokine profiles in AH would affect the graft acceptance and the long-term functional integrity of reconstituted HCE. As shown in Supplementary Fig. 2a, the levels of cytokines in AH are greatly distinctive (more than a hundred times) among individuals. This means that the pre-surgery profiles of cytokines in the AH of nine patients enrolled for the first stage of clinical research may be, at least partly, influential on the functional integrity of the reconstituted HCE (Supplementary Fig. 2b) and imply the importance of personalized medical concerns in the outcome of each individual.

### No graft/host wound: comparison among PK, DSAEK and DMEK

**Figure 5:**
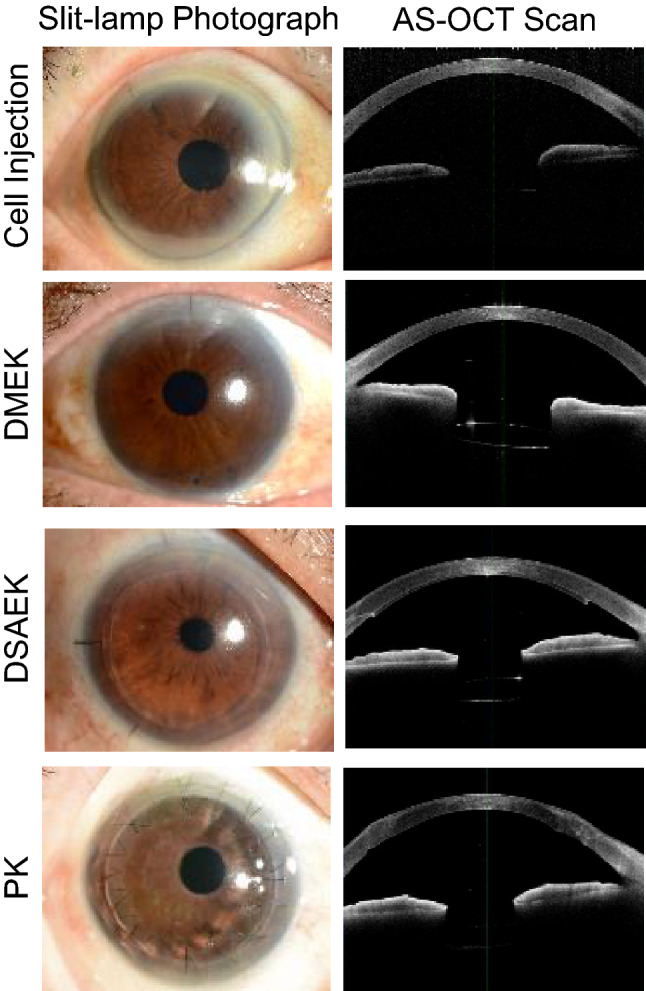
Slit-lamp microscopic images (left) and anterior segment optical coherence tomography (AS-OCT) images (right) of the eyes obtained using CASIA and CASIA2 (Tomey Corporation, Nagoya, Japan) at 6 months postoperative: post-cell injection (top, CASIA), post-Descemet membrane endothelial keratoplasty (DMEK) (second column, CASIA 2), post-Descemet’s stripping automated endothelial keratoplasty (DSAEK) (third column, CASIA 2) and post-penetrating keratoplasty (PK) (Bottom, CASIA 2). The postoperative shape of the cornea receiving cell injection is almost identical to that of a healthy cornea, as well as the eye that underwent DMEK.

So far, the largely disregarded issue is the graft/host wound influence on the local innate inflammatory response. Shaped keratoplasty procedures have allowed corneal surgeons to use complex graft-host junctions and non-circular graft designs to optimize wound healing. Our new surgical modality, hCEC injection, may overcome this issue by generating simple, smooth graft-host junctions and non-circular graft designs, which may be considered beneficial to minimize wound stress and the local innate inflammatory response when compared with those of PK, DSAEK and even DMEK (Fig. 5).

### Induction of immune tolerance did not dampen cyclosporine A

Immunosuppressive drugs, including steroids, may suppress not only the rejection response but also immune tolerance because of the blockade of antigen sensitization. In some experiments, the high-risk rejection model was also established by inducing corneal neovascularization 2 weeks earlier by placing 3 interrupted 11-0 sutures in the central cornea. In these high-risk PK mouse model with neovascularization, most allografts were rejected within 4 weeks in the vehicle group. Topical application of cyclosporine A (CyA) suppressed the rejection, and allografts survived for more than 8 weeks in 70% of mice (Supplementary Fig. 3).

Allograft rejection was also suppressed at the same rate, even when administered in the eye opposite the transplant. In both cases, immune tolerance was induced 8 weeks after transplantation, and no further allograft rejection occurred. It was hypothesized that immunosuppressive drugs can achieve both the suppression of allograft rejection and the induction of immune tolerance.

### Discussion

The most notable medical innovation of our hCEC injection therapy is the reconstitution of functionally healthy transparent CE, even for patients with a history of failed corneal grafts (Fig. 1). The defined rejection was absolutely absent and scanning slit contact specular microscopy showed no features of graft rejection on any of the reconstituted CE, which was carefully inspected throughout the more than 5 years period after surgery^9–11^ (Fig. 1, Table 1).

HCE can impair the functions of infiltrating CD4 + Th1 cells via the PD-1/PD-L1 interaction^25^. GITRL may expand Treg within the cornea to induce immune privilege^26^. We confirmed that the expression of surface HLA class I antigen on hCEC SPs with high clinical efficacy is almost null compared with hCEC SPs with low clinical efficacy (Supplementary Fig. 1)^14^.

To prevent the detrimental outcome of intraocular inflammation, the eye possesses exquisite structures by which the innate and/or adaptive immune-inflammatory axis can be silenced^20–23,27–29^. We actually observed a quite distinct array of cytokine profiles in the pre-surgical AH of the patients enrolled (Supplementary Fig. 2a,b). This encouraged us to investigate the presence or absence of alloantigen-specific ex vivo responses against hCECs in patients. This critical investigation had first become possible because we can successfully adapt, in MLR, hCECs as ex vivo stimulatory allogeneic cells, which bear identical alloantigens with the in vivo injected hCECs. The ocular immune privilege and ACAID^27,30–34^ is responsible for the low risk of CEC transplantation, and the CE is less immunogenic than the corneal stroma and epithelium^15,16,30^. The observations in Fig. 3a,b are in line with our clinical findings that reconstituted CE is less likely to be rejected. Alloantigen-specific DTH was not significantly different from the negative control at 4 weeks after transplantation in all mpCEC-injected mice (Fig. 3a). IFN-γ produced by CD4 + T cells or innate responses may activate macrophages to elevate DTH. In this context, the repressed ELISPOT for IFN-γ (Fig. 2b) implies the absence of DTH activation in patients against alloantigens on injected hCECs, consistent with the observations in murine models^17,24^. Intriguingly, the mouse PK model confirmed that allografted acceptor mice displaying no rejection episodes for 8 weeks certainly acquired donor-specific tolerance (Fig. 3b)^17,35^.

Corneal immune and angiogenic privilege^36^ is mainly dependent on resident heterogeneous immune cells^19,29,37–39^. A systemic immunosuppressive effect of CyA in the high-risk PK mouse model did not interfere with the repression of allo-DTH in a recipient mouse with surviving corneal tissue allograft 8 weeks after transplantation (Supplementary Fig. 3). The necessity of topical application of steroids, such as immune-suppressive and anti-inflammatory drugs, and the timing of discontinuation post-surgery in our cell injection therapy is still a critical issue, and we will also await future research in hCEC injection therapy.

It has been widely accepted that the innate immune–inflammatory axis is critical for the long-term acceptance of the corneal allograft^40–46^ alongside the adaptive immune responses illustrated by DTH and cytokine production by CD4 + T cells. Even in relatively low-risk DMEK patients, critical cytokines of the innate immune system in the AH were elevated in patients undergoing repeated DMEK for graft failure^44^. On a related note, in PK and DSAEK eyes, preoperatively elevated cytokine levels were reportedly associated with primary graft failure^45^. In connection with the diversity of the cytokine milieu in AH, we preliminary found the critical cytokines influencing the long-term survival of corneal tissue allografts, but not in the cell injection therapy (Ueno, unpublished data).

An intriguing but largely ignored issue is the graft/host wound influence, albeit in part, on the local innate inflammatory response, leading to the chronic functional integrity of the corneal allograft. Cell injection therapy generated simple, smooth graft-host junctions and non-circular graft designs, which may be considered to benefit from minimizing the chronic innate immune-inflammatory response. The molecular mechanism underlying in presence of a very small proportion of graft rejection, even in DMEK^1^, may await the future extensive study. In this context, the possible presence of nonvisible minor distortions, regardless of their having the same appearance as those with hCEC injection therapy (Fig. 5), might be one of the working hypotheses worthwhile to be experimentally validated. To define the influence of graft/host wound features on the inflammatory milieu in AH, a precise evaluation of the molecular dynamics of the innate inflammatory axis (cytokines, metabolites, miRNAs and extracellular vesicles) within the AC after surgical intervention is urgently needed.

In 2015, the group of Dana et al.^47^ reported with respect to murine systems that polymegethism (but not pleomorphism or cell density) is a sensitive indicator of the effect of alloimmunity on CECs indicating the relevance of not only adaptive immunity but also innate inflammation. These arguments were consistent with our findings in humans that the clinically efficient hCEC SPs used in our hCEC injection therapy evidently had a narrowed distribution of cell areas and decreased average cell sizes^48^. Additionally**,** it is relevant that the rejuvenation of the CEC layer with a higher level of collective cell order, as evaluated by specular microscopy images at 24 weeks and 3 years post-surgery, showed minimum long-term chronic graft failures^11,49^.

## Methods

### Patients

This small case series clinical trial of hCEC injection therapy using hCECs was approved by the Institutional Review Board and the Specially Certified Committee for Regenerative Medicine of Kyoto Prefectural University of Medicine, Kyoto, Japan (Approval No. RBMR-R-31-4), and by the Special Committee of the Japanese Ministry of Health, Labor and Welfare (MHLW), to observe the guidelines on clinical research using human stem cells in Japan and the act on the safety of regenerative medicine (Approval No. 0329-23) or the Health Science Council of MHLW. Clinical trial registration was obtained at UMIN000012534 (http://www.umin.ac.jp/english/) and jRCTa050190118 (https://rctportal.niph.go.jp/en). Informed consent was obtained from all subjects participating in this study. The protocol of the prospective observational study and that of the laboratory study were approved by the Institutional Review Board of Kyoto Prefectural University of Medicine (Approval No. 1604) (UMIN Clinical Trials Registry No. UMIN000036422) and (Approval No. ERB-C-245-8, ERB-C-584 and ERB-C-978), respectively.

Following surgery, all patients underwent systemic and topical administration of steroids to inhibit acute inflammation and/or immunological reaction in accordance with the drug regimen administered in our regular corneal transplantation procedures (Supplemental Table S2)^9^.

### HCE donors and cell cultures of HCE cells for injection

The human tissue used in this study was handled as detailed in the previous publications^8–10^. Two patients with PK failure and one patient with DSAEK failure had fine neovascularization in the host peripheral cornea, but not in the donor cornea.

### Quantitative analysis of cytokines by Bio-Plex

Patients’ sera were collected on the day before and 2 days, 1 week, 2 weeks and 1 month after hCEC injection and frozen at − 80 °C until analysis. We carefully investigated the patients enrolled as the first 9 subjects in our clinical research. The AHs of BK patients and those of patients underwent cell injection therapy were collected on the day of surgery and stored in the same conditions as the sera samples. The cytokine levels of each sample were analyzed using Luminex Corporation (Austin, TX, USA) xMap Technology (Bio-Plex 200; Bio-Rad Laboratories, Inc., Hercules, CA, USA) with the Bio-Plex Human 27-Plex Panel Kit (Bio-Rad Laboratories) according to the manufacturer’s instructions.

### Flow cytometric analysis

The fully matured hCECs (5 weeks after cell seeding) were collected, and 1 × 10^5^ cells were stained with the following antibodies: Phycoerythrin-conjugated anti-CD40L mAb (Abcam, Cambridge, UK), APC-conjugated anti-B7-H1/PD-L1 mAb (Thermo Fisher Scientific), Phycoerythrin-conjugated anti-Thrombospondin 1 Antibody mAb (Santa Cruz Biotechnology, Santa Cruz, CA), APC-conjugated anti-Human CD152 (CTLA4) mAb (BD Pharmingen, San Jose, CA), Phycoerythrin-conjugated anti-GITR Ligand/TNFSF18 mAb (R&D systems, Minneapolis, MN), Phycoerythrin-conjugated anti-TGF-beta RII mAb (R&D systems), biotinylated anti-CD178 (FasL) mAb (Thermo Fisher Scientific) and Streptavidin APC (Thermo Fisher Scientific), FITC-conjugated anti-CD26 mAb (BD Pharmingen), PerCP-Cy5.5-conjugated anti-CD44 mAb (BD Pharmingen) and Alexa Fluor® 647-conjugated anti-HLA mAb (Santa Cruz Biotechnology). The hCECs were analyzed using a BD FACSCanto II Flow Cytometry System (BD Biosciences, San Jose, CA, USA).

### ELISPOT assay

ELISPOT assays were performed using the Human IFN-γ ELISpot Pro assay kit (Mabtech, Sweden) according to the manufacturer’s instructions. Fifteen and 12 patients were enrolled to the government-approved doctor-initiative phase II and phase III clinical trials of hCEC injection respectively. The phase II clinical trial was to determine the appropriate dose of cells for injection, and the phase III clinical trial was to confirm its safety and efficacy. Thus, the amounts of injected cells for each patient were 0.2–1.0 × 10^6^ cells in the phase II trial and 1.0 × 10^6^ cells in the phase III trials. Patients were randomly selected without any bias for the immunological analysis. Peripheral blood was collected from 7 of 15 phase II patients and 10 of 12 phase III patients on the day before and 2 weeks after the hCEC injection therapy. Peripheral blood mononuclear cells (PBMCs) of the patients were isolated from heparinized whole blood using Lymphoprep™ density gradient medium (Stem cell technologies, Vancouver, BC, Canada). PBMCs (2.5 × 10^5^ cells/well) were mixed (stimulated) with hCECs from the same batch (i.e. the same donor) as those injected into the patients or PBMCs from normal donors (5 × 10^4^ cells/well) and incubated for 2 days. After washing the wells with PBS (-), IFN-γ positive spots were detected and counted with an ELISPOT reader (Autoimmun Diagnostika GmbH, Germany). To examine the optimal stimulator/responder (S/R) ratio, PBMCs from normal donors (2.5 × 10^5^ cells/well) were mixed with hCECs (2.5 × 10^5^ cells/well [S/R ratio of 1/1] or 5 × 10^4^ cells/well [S/R ratio of 1/5]) and the IFN-γ-specific spots were detected as described above.

### Animal experiments

All methods were performed in accordance with the relevant guidelines and regulations. For animal experiments we had followed the approval of the Experimental Animals Committee, Kyoto Prefectural University of Medicine, including any relevant details and all experiments were performed in accordance with relevant guidelines and regulations. Furthermore, the reporting in the manuscript follows the recommendations in the ARRIVE guidelines.

Male BALB/c (H-2d) mice (SLC, Osaka, Japan) between 8 and 12 weeks of age were used. All animals were treated according to the Association for Research in Vision and Ophthalmology Statement for the Use of Animals in Ophthalmic and Vision Research. All experiments were approved by the Committee for Animal Research of Kyoto Prefectural University of Medicine and performed according to the method described previously, including preparation of murine primary corneal endothelial cells (mpCECs), the injection of mpCECs (1 × 10^4^ cells in 3 μL HBSS) into AC and the assessment of DTH and ACAID induction^23^.

### Orthotopic corneal transplantation and assessment of graft rejection

To determine the capacity of the recipient injected with mpCECs to reject allogeneic mpCECs, we transplanted healthy C57BL/6 corneas onto the eyes of BALB/c mice that had been previously injected with C57BL/6 mpCECs into the AC. As for the mpCECs injection, after eliminating the BALB/c endothelial cells by cryoinjury, collected C57Bl/6 mpCECs (1 × 10^4^ cells in 3 μL HBSS) were injected into AC through an oblique incision without any leakage^23^. For the orthotopic corneal transplantation control, healthy C57BL/6 corneas were transplanted onto the eyes of BALB/c mice. In some experiments, the high-risk rejection model was also performed by inducing corneal neovascularization 2 weeks earlier by placing 3 interrupted 11-0 sutures in the central cornea. The corneal transplantation technique has been described elsewhere^34,49^. All grafts were examined twice a week with a slit-lamp biomicroscope. At each time point, grafts were scored from 0 to 5+ for opacification, using a previously described scoring system^34,49^. Grafts exhibiting an opacity score of 3+ or greater (moderate stromal opacity with only the pupil margin visible) at 2 weeks, or 2+ or greater (mild deep [stromal] opacity with the pupil margin and iris vessels visible) after 3 weeks, were considered rejected.

### Statistical analysis

Student’s paired t-test was used to compare corneal thickness. *p*-values of < 0.05 were considered statistically significant.

Kruskal–Wallis test was used to compare corneal thickness and, if statistically significant differences were found, post‐hoc analysis with Steel's multiple comparison test. *p*-values of < 0.05 were considered statistically significant. The statistical analyses above were performed using R v.4.1.2 (The R Foundation for Statistical Computing, Vienna, Austria).

## Supplementary Information

Supplementary Information.

## Data Availability

All data generated or analysed during this study are included in this published article and its Supplementary Information files. Further data are available from the corresponding author on reasonable request.
