# Supplementary material for: Quiescent innate and adaptive immune responses maintain the long-term integrity of corneal endothelium reconstituted through allogeneic cell injection therapy
Source: Sci Rep. 2022 Oct 27;12:18072. doi: 10.1038/s41598-022-22522-4 (PMC9613641; doi:10.1038/s41598-022-22522-4)
Supplement: Supplementary file 1 — Supplementary Information. [file 41598_2022_22522_MOESM1_ESM.pdf]

Quiescent Innate and Adaptive Immune Responses Maintain the Long-Term Integrity of Corneal Endothelium Reconstituted through Allogeneic Cell Injection Therapy

Munetoyo Toda,[1] Morio Ueno,[2] Jun Yamada,[2] Asako Hiraga,[2] Kazuko Asada,[2] Junji Hamuro,[2,*] Chie Sotozono,[2] and Shigeru Kinoshita[1]

[1]Department of Frontier Medical Science and Technology for Ophthalmology, Kyoto Prefectural University of Medicine, Kyoto, Japan

[2]Department of Ophthalmology, Kyoto Prefectural University of Medicine, Kyoto, Japan

*Correspondence: Junji Hamuro, Ph.D., Department of Ophthalmology, Kyoto Prefectural University of Medicine, 465 Kajii-cho, Hirokoji-agaru, Kawaramachi-dori, Kamigyo-ku, Kyoto 602-0841, Japan.

Tel: +81-75-251-5204; Fax: +81-75-251-5663; E-mail: jshimo@koto.kpu-m.ac.jp

Supplemental Information

Supplementary Table 1

Specific cytokines are elevated in aqueous humors of CEF patients

Analyte	median	min	max
IL-1ra	28.75	0	769.75
IL-6 *	275.58	5.44	119653.08
IL-7	5.93	0	24.21
IL-8 *	69.23	5.78	1116.97
IL-10	7.43	2.62	17.86
IL-12 (p70)	10.45	0	41.26
IL-15	21.66	0	100.38
FGF basic	25.43	0	90.15
Eotaxin	10.38	0	210.83
G-CSF *	44.30	0	9921.37
GM-CSF *	406.781	71.24	781.72
IFN-γ	10.38	0	210.83
IP-10 *	452.05	37.22	12179.16
MCP-1 *	1117.91	271.88	4984.00
MIP-1β	26.86	4.67	99.18
RANTES	7.29	0	55.71
TNF-a	5.23	0	189.70
VEGF	88.92	1.84	342.34

The AH of CEF patients were collected on the day of surgery and stored frozen at -80°C until analysis. The cytokine levels of each sample were analyzed as described in the Materials and Methods section. *Higher than the published data with the AH of patients with diabetes mellitus.[1]

1. Dong, N., Xu, B., Wang, B., & Chu, L. Study of 27 aqueous humor cytokines in patients with type 2 diabetes with or without retinopathy. *Mol Vis*. **19,** 1734-1746 (2013).

Supplementary Table 2

Drug Administration Regimen

Route	Drug Type	Drug Name	Dose	3-days pre-op	day of surgery	1-2 days post-op	3-days post-op	4-7 days post-op	Up to 24-weeks post-op	Up to 2-years post-op
Local Admin.	Antibiotics	Gatifloxacin hydrate	4 times per day	■	■	■	■	■	■	■
	Steroids	Beta-methasone	4 times per day	■	■	■	■	■	■	
		Fluoro-metholone	2-4 times Per day							■
Systemic Admin.	Antibiotics*	Flomoxef Sodium	1g per day		■	■				
		Cefcapene pivoxil hydrochloride	3 tablets		■	■				
	Steroids	Methyl-prednisolone	125 mg i.v.		■	■				
		Beta-methasone	2 mg i.v.			■	■	■		
		Beta-methasone	1 mg					■		

Admin.; administration, i.v.; intravenous administration, op; operation, *Administration of either Flomoxef sodium or Cefcapene pivoxil hydrochloride

This table was adapted from the supplementary appendix of reference 8.

Supplementary Figure 1

Expressions of immunomodulatory molecules on cultured hCECs.

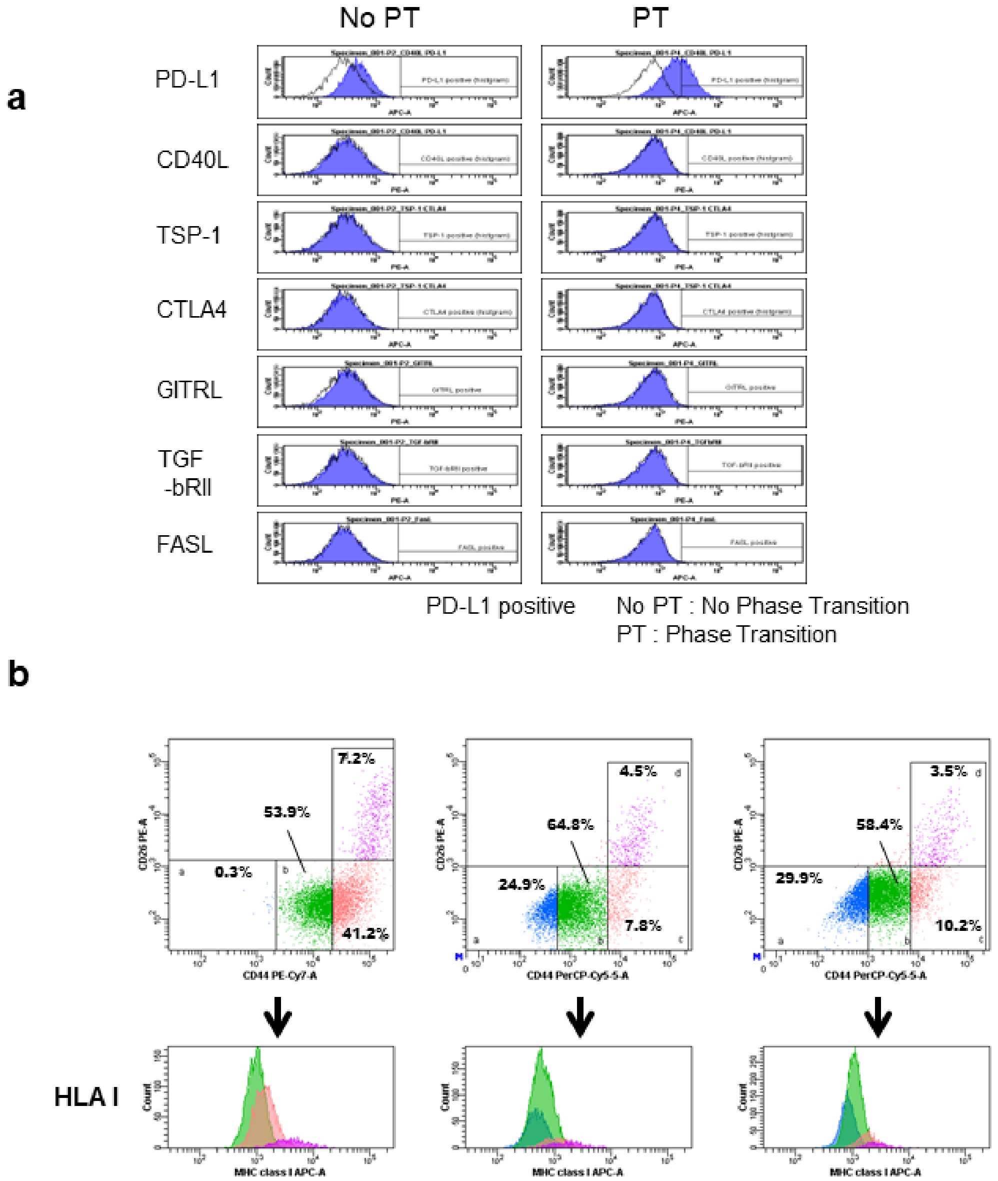

Expressions of immunomodulatory molecules on cultured hCECs. (A) Cultured hCECs at P0 were labeled with each specific antibody and analyzed using a flow cytometer（N=3）. (B) Three different lots containing different hCEC SPs injected to patient #1~3 in Table1 were analyzed using a flow cytometer. The cells were assigned to four SPs by the expression of CD26 and CD44, and then the HLA class I expressions of each SP were evaluated.

Supplementary Figure 2

Variances among individuals in terms of cytokines in AH

a

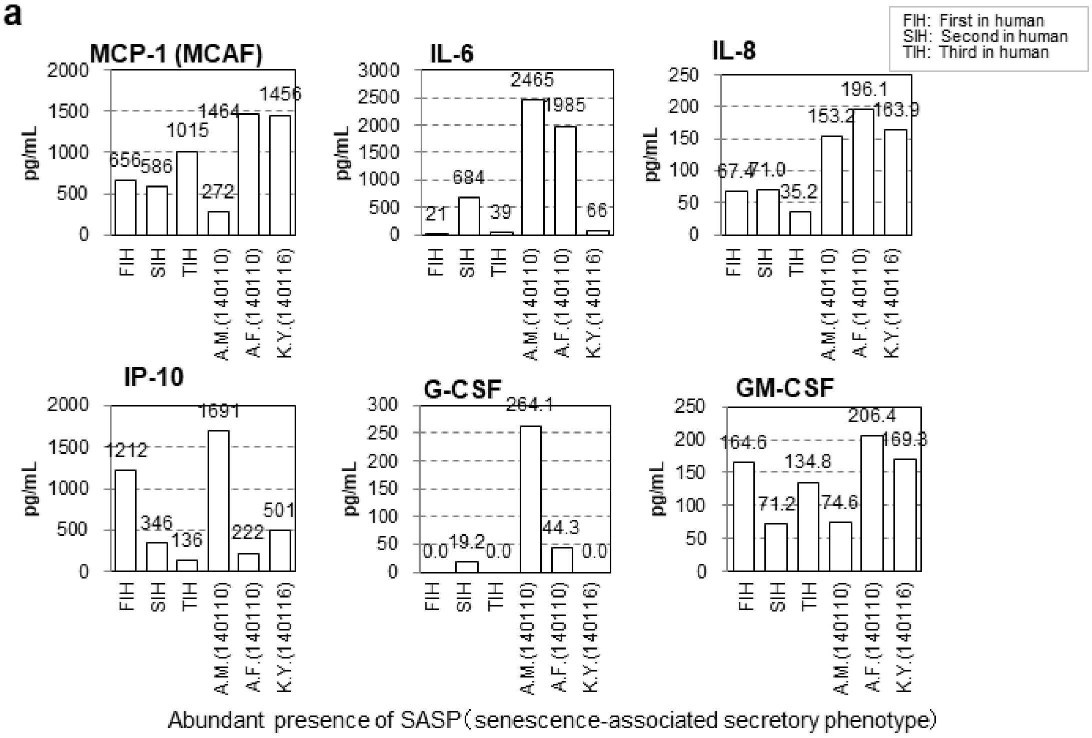

b

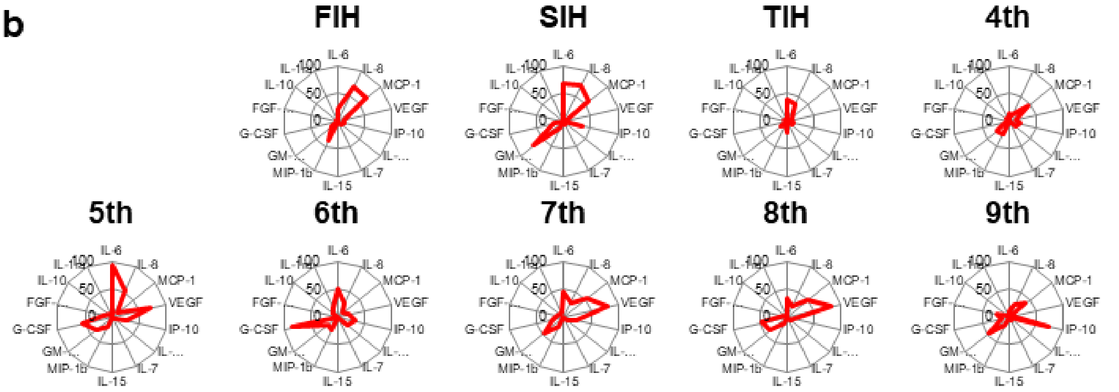

Variances among individuals in terms of cytokines in AH. AH from BK patients were collected on the day before surgery and analyzed using Bio-Plex. (A) Bar graphs of cytokine expression in the 1st to 3rd patients who received cell infusion therapy in the clinical research and of three patients who received traditional keratoplasty. (B) Radar charts of the cytokine profiles in the AH of nine patients who received first-in-human to 9th therapy in the clinical research.

Supplementary Figure 3

Induction of immune tolerance is not dampened by cyclosporine.

After Transplantation	Vehicle	CyA	CyA (fellow eye)
2 wks	60%	100%	100%
4 wks	10%, DTH^{+}	80%, DTH^{-}	80%, DTH^{+}
8 wks	0%	*70%, DTH^{-}	*70%, DTH^{-}

*Induction of tolerance

CyA : Cyclosporin A

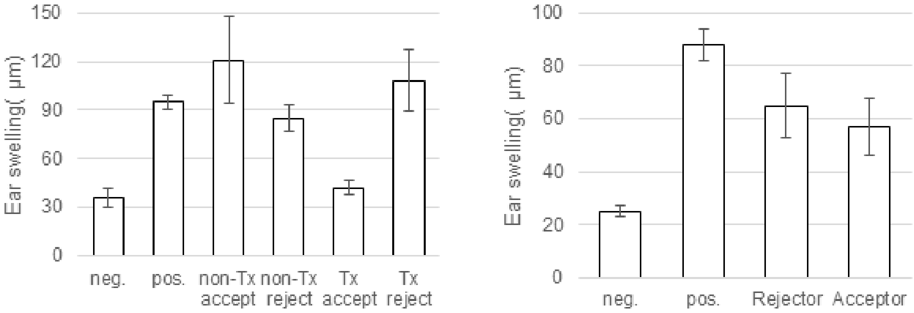

The induction of immune tolerance is not dampened by cyclosporine. A. Neovascularized high-risk eyes were prepared in both eyes of BALB/c mice. The right eyes received C57BL/6 corneal allografts and the left eyes received BALB/c autografts with 0.1% cyclosporine A eyedrop treatments three times a day for four weeks. The systemic effects of CyA never inhibited corneal allograft-induced tolerance eight weeks postoperatively and suppressed corneal allograft rejection (n = 10 each, Student’s paired t-test； $p < 0.001$). Further, Kruskal‐Wallis test and post‐hoc analysis with Steel's multiple comparison test (lower left figure) were applied. Kruskal‐Wallis test P<0.00, Steel's multiple comparison test; Neg vs Pos P=0.039, Neg vs non-Tx Acceptor P=0.197, Neg vs non-Tx rejector P=0.015, Neg vs Tx accept P=0.996, Neg vs Tx reject P=0.060, (lower right figure) Kruskal‐Wallis test P=0.006, Steel's multiple comparison test; Neg vs Pos P=0.025, Neg vs Rejector P=0.039, Neg vs Acceptor P=0.044
